# Editorial: Stowaways of a Stowaway: Parasites of Invasive Rodents

**DOI:** 10.3389/fvets.2022.844511

**Published:** 2022-02-21

**Authors:** Alexis Ribas, Heikki Henttonen, Serge Morand

**Affiliations:** ^1^Parasitology Section, Department of Biology, Healthcare and Environment, Faculty of Pharmacy and Food Science, IRBio (Research Institute of Biodiversity), University of Barcelona, Barcelona, Spain; ^2^Wildlife Ecology, Natural Resources Institute Finland, Helsinki, Finland; ^3^MIVEGEC, CNRS, IRD, Montpellier University, Montpellier, France; ^4^Faculty of Veterinary Technology, Kasetsart University, Bangkok, Thailand

**Keywords:** rodent, zoonosis, invasion, parasite, *Rattus*, *Mastomys*, *Mus*

Human-induced ecosystem modifications include the introduction of alien species, among which rodents are particularly widespread. The expansion and adaptations of invasive rodents, which can come from distant regions, even different continents, have a wide spectrum of ecological consequences, including the spread of parasites and pathogens. As the title of this Research Topic reflects, the parasites travel to these newly colonized areas as stowaways on a stowaway (1, 2).

Studies of parasites of invasive rodents have focused mainly on those carried by the genera *Rattus* and *Mus*. The available literature describes the parasites found and their prevalence, with particular attention given to zoonotic species that pose a threat for human health, and analyses of the specific host traits that enable successful invasions. Some recent studies have also investigated the spillover effect, shedding light on how the stowaway parasites are transmitted to native rodent species, and the role played by both invasive and native animals in transmission dynamics and maintenance of the new parasites in colonized environments. Our aim here is to focus on recent work in the field, dealing with previously unexplored Research Topics, and to identify research gaps and new questions to be pursued in the future.

To provide a global view of the impact of invasive rodent parasites, the contributions selected for this Research Topic include field studies carried out in Africa, Asia, and Europe, as well as a review paper focusing on South America. An advantage of working with rodents as model taxa is the relative facility of obtaining sufficient replicates, yet despite this many studies on rodents are limited by modest sample sizes, as more extensive sampling requires intensive field work and laboratory time. This is not the case in the studies included here: Broecke et al. analyzed more than 200 rodents, an exceptionally high number for the kind of behavioral study they performed; Herrero-Cófreces et al. summarize 10 years of trapping data; the work of Obiegala et al. involved the screening of over a thousand fleas, and Wu et al. studied an astounding 105,680 chiggers.

Studies in this field have mainly focused on agricultural land, the favored habitat of urban rodents (*Rattus* and *Mus*) in their new distribution areas. Our aim was to cover a wider range of rodents and ecosystems: Obiegala et al. explore *Bartonella* infection in small mammals from three structured sites (urban, sylvatic, and renatured), and Wu et al. compare grassland and forest sampling sites.

Transmission pathways are better understood when the different trophic levels involved are analyzed in their full complexity. Multihost systems are well-represented here: Obiegala et al. describe a three-level parasite network (parasite-vector-host), involving five species of *Bartonella* in 12 species of fleas and 10 small mammal species. Wu et al. also explore a tripartite parasite system: the rickettsia *Orientia tsutsugamushi*—trombiculid mites—Asian house rat (*Rattus tanezumi*); Julius et al. analyze the distribution of cestodes in three alien *Rattus* species and the indigenous *Mastomys coucha;* and Diagne et al. report parasites in invasive *Mus* species and the native *Mastomys erythroleucus* and *Arvicanthis niloticus*. In their study of *Mastomys natalensis* and its helminths, Broecke et al. add complexity by assessing individual behavior. We are still far from establishing a solid knowledge of the relationship between animal behavior and parasites. In this context, Broecke et al. obtained unexpected results, finding that less explorative individuals had a higher infection probability with a greater diversity of helminths.

Also discussed are the parasites that rodents can transmit to humans. Obiegala et al. report on the bacteria *Bartonella grahamii*, Wu et al. focus on *O. tsutsugamushi*, the causal agent of scrub typhus, and Herrero-Cófreces et al. look at how changes in farming practices have affected the dynamics of rodents and Francisella *tularensis*, the bacterium responsible for tularemia. Broecke et al. and Julius et al. discuss potential zoonotic helminths, and Alcover et al. review the risk of the protozoan *Leishmania infantum* in rodents. Completing this spectrum of parasites, a review about Landaeta-Aqueveque provides a detailed picture of macro-parasites in native and invasive rodents in Chile.

In *Stowaways on a Stowaway: Parasites of Invasive Rodents*, we provide a broad perspective on invasive rodents and identify future research challenges. A more in-depth evaluation of the role of rodents in parasite transmission to livestock, domestic animals, and humans is necessary. Despite considerable new knowledge regarding parasite transmission between native and invasive rodents, a holistic One Health approach will provide a better understanding of pathogen transmission processes. Future studies should include both past and recent invasions, and as these invasive processes are of global concern, all biogeographical areas are worthy of research.

## Author Contributions

AR wrote the first draft of the manuscript. All authors contributed to the editorial revision, read, and approved the submitted version.

## Conflict of Interest

The authors declare that the research was conducted in the absence of any commercial or financial relationships that could be construed as a potential conflict of interest.

## Publisher's Note

All claims expressed in this article are solely those of the authors and do not necessarily represent those of their affiliated organizations, or those of the publisher, the editors and the reviewers. Any product that may be evaluated in this article, or claim that may be made by its manufacturer, is not guaranteed or endorsed by the publisher.
